# Supplementary material for: Photoinitiated Polymerization of Hydrogels by Graphene Quantum Dots
Source: Nanomaterials (Basel). 2021 Aug 25;11(9):2169. doi: 10.3390/nano11092169 (PMC8470854; doi:10.3390/nano11092169)
Supplement: Supplementary file 1 [file nanomaterials-11-02169-s001.zip › nanomaterials-1343413-supplementary.pdf]

nanomaterials

MDPI

Supplementary Materials

# Photoinitiated Polymerization of Hydrogels by Graphene Quantum Dots

**Yuna Kim [1,2,†], Jaekwang Song [1,2,†], Seong Chae Park [3,†], Minchul Ahn [1,2], Myung Jin Park [1,2], Sung Hyuk Song [4], Si-Youl Yoo [5], Seung Gweon Hong [5] and Byung Hee Hong [1,2,*]**

[1] Department of Chemistry Seoul National University, Seoul 08826, Korea; ykim2189@gmail.com (Y.K.); saver04@snu.ac.kr (J.S.); mincheol@snu.ac.kr (M.A.); hanson2525@gmail.com (M.J.P.)
[2] Graphene Research Center, Advanced Institute of Convergence Technology, Suwon 16229, Korea
[3] Program in Nano Science and Technology, Graduate School of Convergence Science and Technology, Seoul National University, Seoul 08826, Korea; pscdaniel@snu.ac.kr
[4] Department of Mechanical Engineering, Seoul National University, Seoul 08826, Korea; shksong01023@gmail.com
[5] Interojo Inc., Pyeongtaek 17744, Korea; yoosy@interojo.com (S.-Y.Y.), steve.hong@interojo.com (S.G.H.)
* Correspondence: byunghee@snu.ac.kr
† These authors contributed equally to this paper.

## Dynamic Light Scattering Analysis for Hydrogel

Dynamic light scattering (DLS) analysis is an effective analysis tool for the various relaxation processes in gelation systems. From DLS experiments, the normalized intensity-time correlation function is given by

$$g_2(\tau) = \frac{< I(q,0)I(q,\tau) >}{< I(q,0) >^2} \quad \text{(S1)}$$

where $g_2(\tau)$ is the intensity correlation function, I(q,τ) is the scattered intensity at delay time τ, and q is the scattering wave vector, (4πn/λ)sin(θ/2). Also, the intensity correlation function $g2(\tau)$ is related to the dynamic structure factor S(q,τ) (Equation S2)

$$g_2(\tau) = 1 + \beta'\{2Y(1-Y)S(q,\tau) + Y^2|S(q,\tau)|^2\} \quad \text{(S2)}$$

where $\beta'$ is the coherence area factor, Y is a parameter which provides the measure for the extent of heterodyne contribution. In the sol state, Y is equal to 1, which indicates that the system dynamics can be attributed to purely thermally fluctuating components. Thus, the Siegert relationship in the sol state can be expressed as

$$g_2(\tau) = 1 + \beta'|S(q,\tau)|^2 \quad \text{(S3)}$$

However, in the gel state, Y is less than 1, and the contribution of $2Y(1-Y)$ in equation S2 is dominant. $\sigma^2$, the initial amplitude of $g_2(\tau)$ when S(q,τ)=1 can be expressed as

$$\sigma^2 = Y(2-Y) \quad \text{(S4)}$$

During ergodic (sol) to non-ergodic (gel) transition, gelation factor κ can be obtained by

$$\kappa = 1 - \left|\frac{S_{Et}(q,0) - S_{Et}(q,\infty)}{S_{Et=0}(q,0) - S_{Et=0}(q,\infty)}\right| \quad \text{(S5)}$$

where the term Et is the evolution time after the temperature quench.

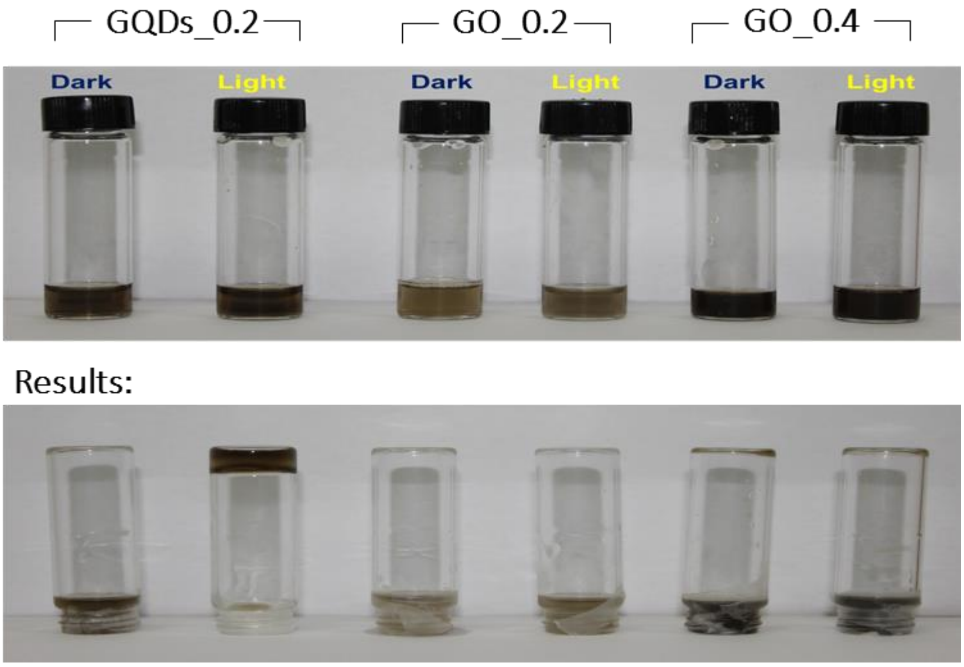


**Figure S1.** Photograph of hydrogel solution mixed with GQDs or graphene oxide before (top)/after (bottom) incubation in dark room or under sunlight. Incubation time in dark room was 24 hours, while incubation under sunlight was 20 min. GO_0.2 and GO_0.4 are the gelation solutions with 0.2 and 0.4 mL of graphene oxide solution (0.01 g/mL), respectively, instead of graphene quantum dots.

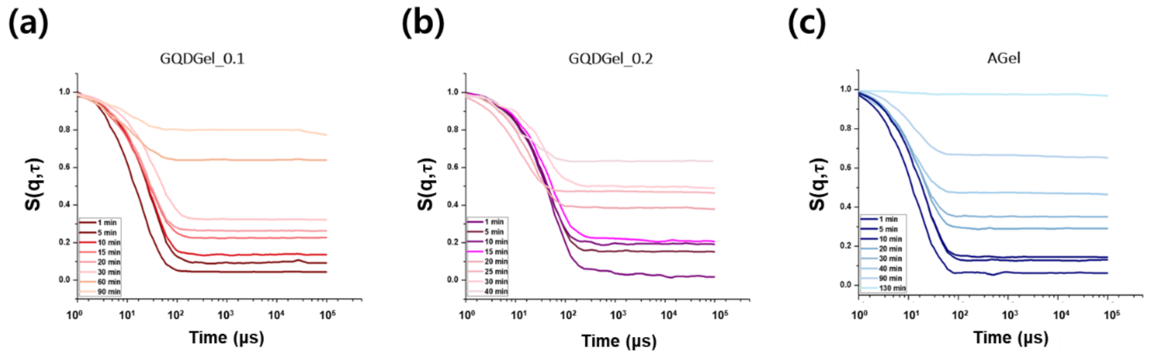


**Figure S2.** Derived dynamic structure factor of hydrogel samples. (**a**) GQDGel_0.1, (**b**) GQDGel_0.2, (**c**) AGel.

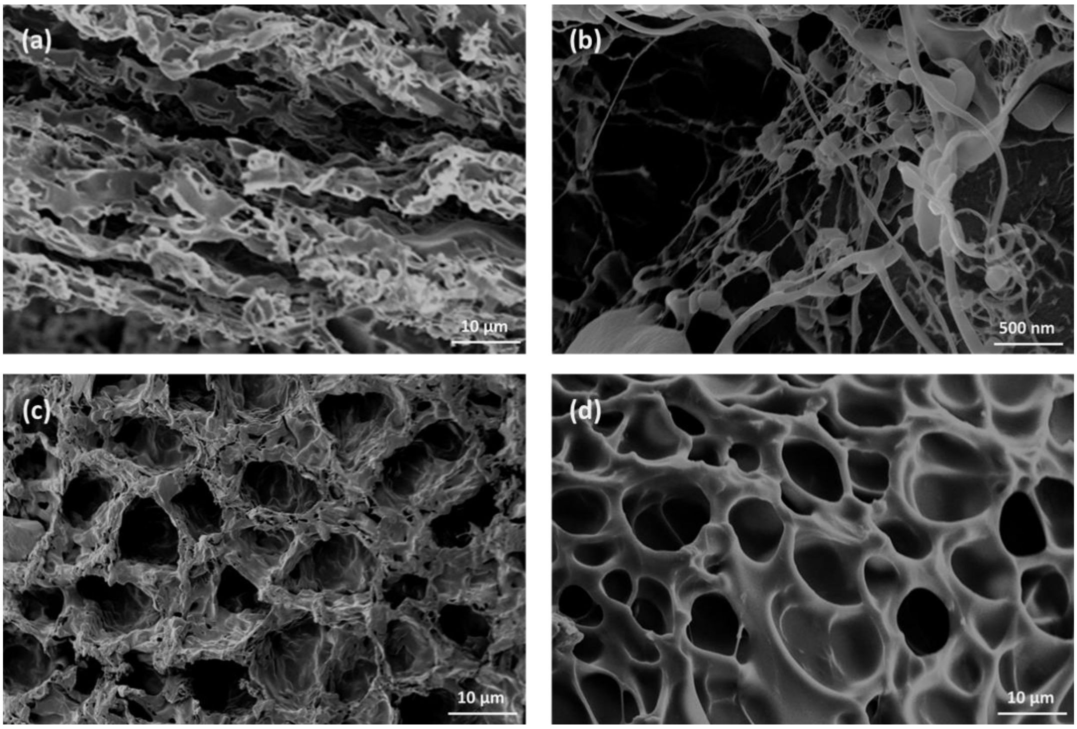


**Figure S3.** Scanning electron microscopy images of GQDGel_0.4 by exposure time. (**a**) 0 min (no irradiation) (**b**) 5 min, (**c**) 15 min, and (**d**) 30 min.

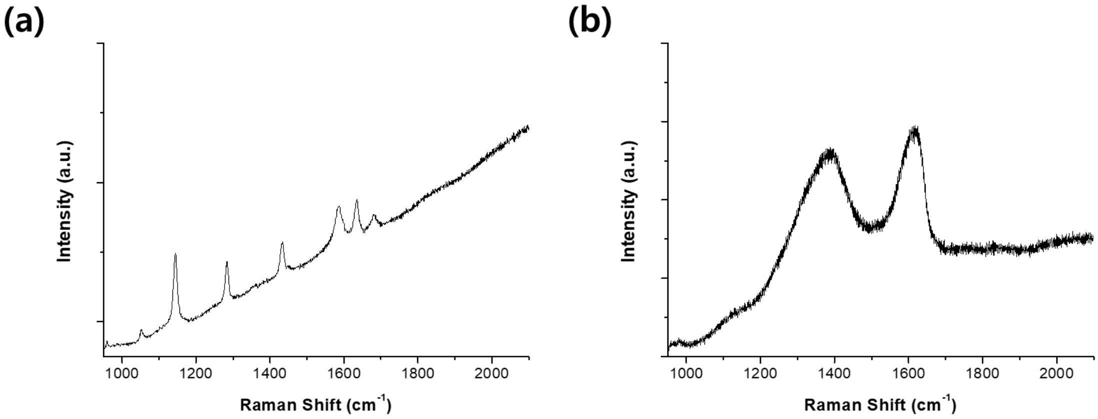


**Figure S4.** Raman spectra of (**a**) as-synthesized GQDGel and (**b**) GQDGel after post treatment. Post treatment is dissolving GQDGel by hydrogen peroxide solution, followed by dialysis and lyophilization to isolate GQDs from GQDGel.

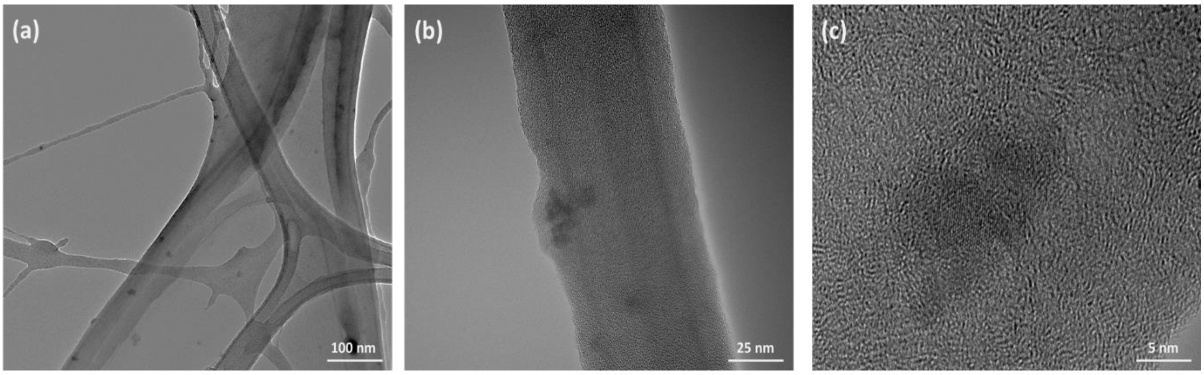


**Figure S5.** (**a**–**c**) Transmission electron microscopy images of the GQDGel_0.1 sample with various magnifications.

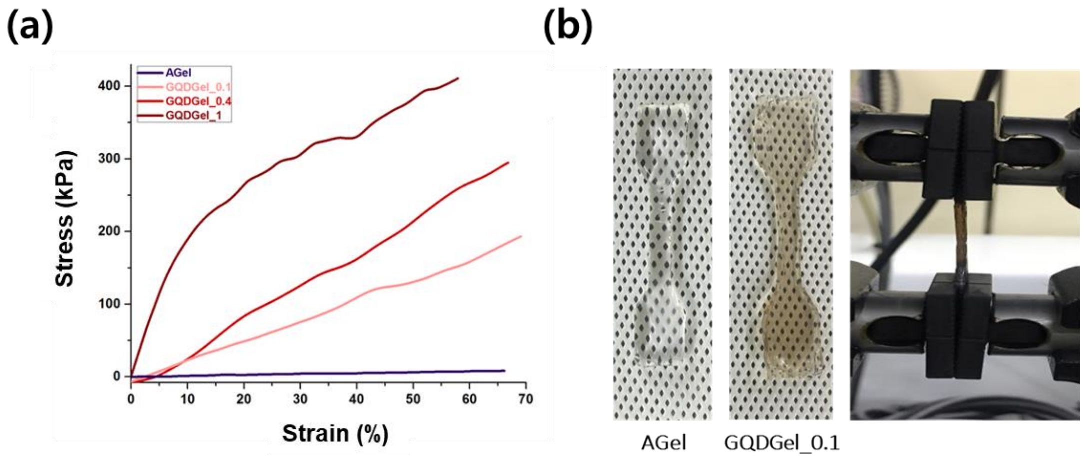


**Figure S6.** Mechanical test for hydrogel samples. (**a**) Stress-strain curve for each sample. (**b**) Photograph of dog-bone shaped hydrogel specimen (left) and clamped specimen during the tensile testing process (right).

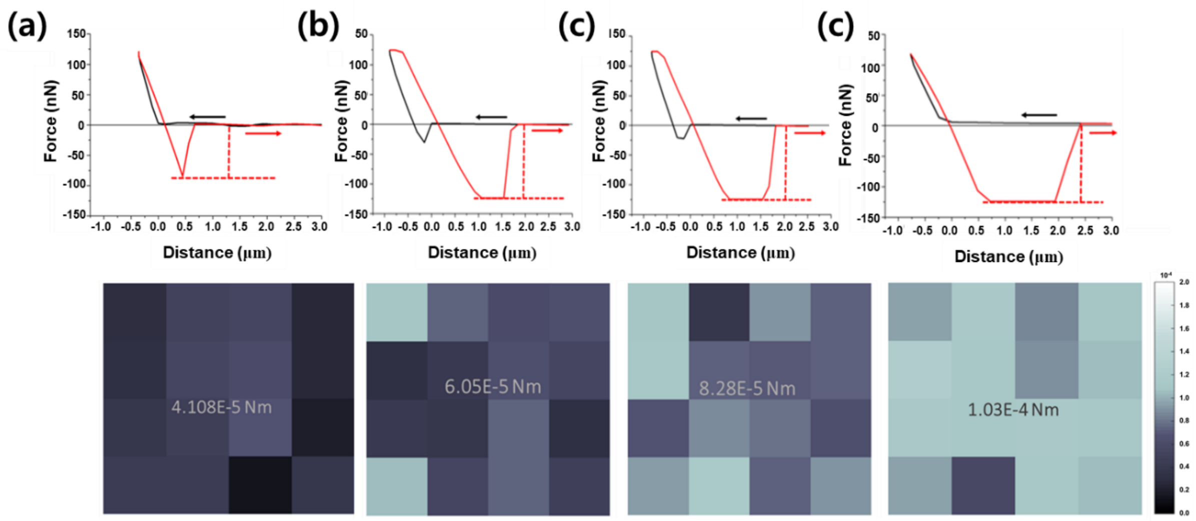


**Figure S7.** Surface adhesion energy measurement of hydrogel samples. (**a**) AGel, (**b**) GQDGel_0.1, (**c**) GQDGel_0.2, and (**d**) GQDGel_0.4 measured by AFM with the contact mode. The upper curves are the force deformation curve of cantilever: approach (black line) and retraction (red line). The bottom mapping is the adhesion force map of hydrogel samples dimension 10 μm x 10 μm. Number in the middle is the average of the adhesion force measured from mapping.

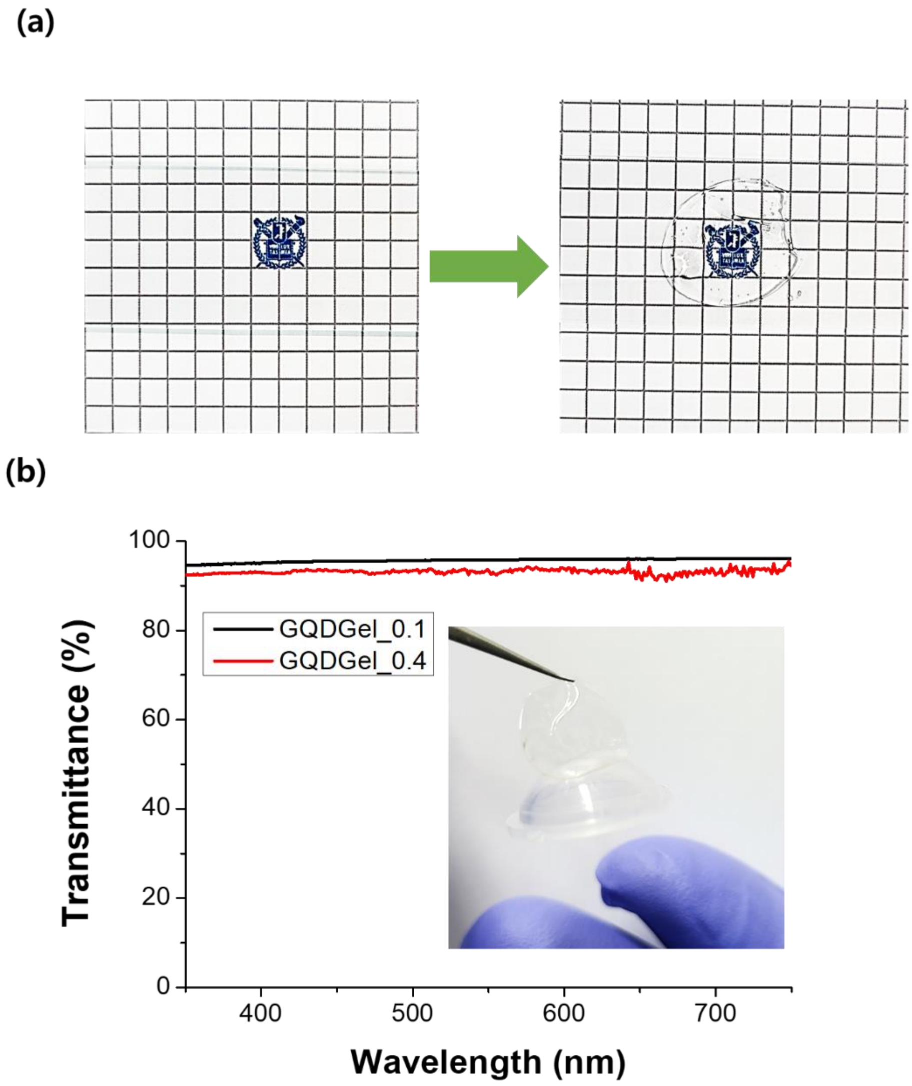


**Figure S8.** Application of GQDGel in the simple contact lens. (**a**) Photographs of GQDGel_0.4 as a simple lens with the thickness of 0.1 mm. Left: without hydrogel lens, right: with hydrogel lens. (**b**) Transmittance of GQDGel_0.1 and GQDGel_0.4 in the visible range. The sample was polymerized in the contact lens mold with the thickness of 0.1 mm. (Inset: a photograph of polymerized lens in the mold for transmittance measurement)**.**
